# Rv0613c/MSMEG_1285 Interacts with HBHA and Mediates Its Proper Cell-Surface Exposure in Mycobacteria

**DOI:** 10.3390/ijms19061673

**Published:** 2018-06-05

**Authors:** Romain Veyron-Churlet, Vincent Dupres, Jean-Michel Saliou, Frank Lafont, Dominique Raze, Camille Locht

**Affiliations:** Université de Lille, CNRS UMR8204, INSERM U1019, Centre d’Infection et d’Immunité de Lille, Institut Pasteur de Lille, 59000 Lille, France; vincent.dupres@ibl.cnrs.fr (V.D.); jean-michel.saliou@pasteur-lille.fr (J.-M.S.); frank.lafont@ibl.cnrs.fr (F.L.); dominique.raze@ibl.cnrs.fr (D.R.)

**Keywords:** tuberculosis, *Mycobacterium*, host-pathogen interaction, adhesin, cell wall

## Abstract

Heparin-binding haemagglutinin (HBHA) is a surface-exposed virulence factor of *Mycobacterium tuberculosis* and is involved in the binding of mycobacteria to non-phagocytic cells, allowing for extra-pulmonary dissemination of the bacilli. Despite its surface exposure, HBHA is not produced as a pre-protein containing a typical cleavable N-terminal signal peptide and is thus likely secreted by a Sec-independent, as of yet unknown mechanism. Here, we used the bacterial adenylate cyclase two-hybrid system to identify the proteins encoded by *rv0613c* and *mmpL14* as being able to interact with HBHA. Our study was focused on Rv0613c, as it showed more consistent interactions with HBHA than MmpL14. Deletion of its orthologous gene *MSMEG_1285* in recombinant *Mycobacterium smegmatis* producing HBHA from *M. tuberculosis* resulted in the loss of proper surface exposure of HBHA, as evidenced by atomic force microscopy. Furthermore, the lack of *MSMEG_1285* also abolished the clumping phenotype and rough colony morphology of the recombinant *M. smegmatis* and reduced its adherence to A549 epithelial cells. These phenotypes have previously been associated with surface-exposed HBHA. Thus, MSMEG_1285 is directly involved in the proper cell-surface exposure of HBHA. These observations identify MSMEG_1285/Rv0613c as the first accessory protein involved in the cell surface exposure of HBHA.

## 1. Introduction

Tuberculosis (TB) remains the leading global cause of infection-related mortality and morbidity, with 1.7 million deaths and an estimated incidence of 10.4 million cases in 2016 [1]. TB pathology is linked to the tight interplay between the human immune system and the persistence of the bacilli within the host. *Mycobacterium tuberculosis* (*Mtb*), the causative agent of human TB, produces virulence factors that contribute to its successful colonization of the host cells. Among them, the Heparin-binding haemagglutinin (HBHA) is a surface-exposed mycobacterial adhesin [2,3] that is able to bind heparan sulfate [4]. It is not involved in the interaction with macrophages but is necessary for the mycobacterial interactions with type II alveolar epithelial cells in vitro [5], and heparan sulfate moieties on proteins, such as Syndecan-4, are implicated in mycobacterial internalization by lung epithelial cells [6]. Although HBHA is dispensable for initial pulmonary colonization, the protein is required for extrapulmonary dissemination of the bacilli in mice [5]. It is a highly methylated protein in its C-terminal part [7] and its complex methylation profile plays a direct role in the immunogenicity and recognition of HBHA by T cells from *Mtb*-infected subjects [8]. An orthologous protein is also present in fast-growing and non-pathogenic *Mycobacterium smegmatis* (*Msmeg*). However, HBHA_*Msmeg* is not involved in epithelial cell adhesion and does not display high affinity for heparin [9]. Thus, HBHA most likely underwent a functional divergence during evolution [9,10]. Despite its cell surface exposure in *Mtb* [2], HBHA lacks a canonical signal peptide, usually required for protein transport through bacterial plasma membranes. Thus, HBHA crosses the mycobacterial membrane by an unknown mechanism that is independent of the classical signal sequences peptides.

In this study, as an approach to unravel HBHA cell surface exposure, we identified the proteins encoded by *rv0613c* and *mmpL14*, as the first proteins able to interact with HBHA. Recombinant *Msmeg* mc^2^155 expressing *hbhA_Mtb* was found to auto-aggregate, to grow as rough colonies and to infect epithelial cells, whereas the deletion of *MSMEG_1285*, the orthologous gene of *rv0613c* in *Msmeg* mc^2^155, abolished these phenotypes. Furthermore, HBHA_*Mtb* was detected by atomic force microscopy at the surface of recombinant *Msmeg* mc^2^155 but not of the recombinant Δ*MSMEG_1285* mutant, indicating an important role for Rv0613c/MSMEG_1285 in the cell-surface exposure of HBHA.

## 2. Results

### 2.1. The N-Terminal Part of HBHA Interacts with Rv0613c and MmpL14

**Figure 1:**
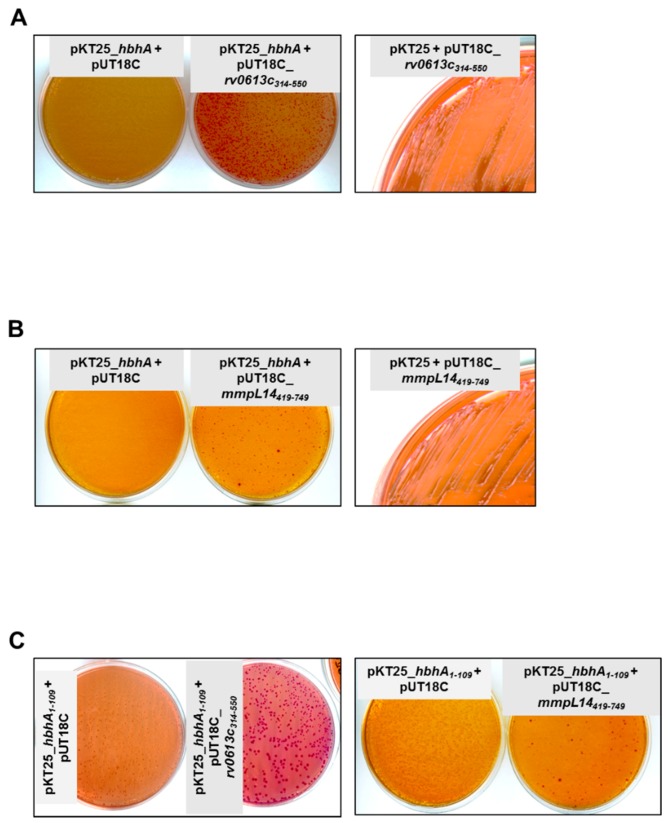
HBHA interacts with Rv0613c and MmpL14 in the BACTH system. (**A**) Red colony phenotype observed on MacConkey agar plates for *E. coli* transformed with pKT25_*hbhA* and pUT18C_*rv0613c*_314–550_ (middle plate) in contrast to the *E. coli* transformed with pKT25_*hbhA* and pUT18C (left plate) or pKT25 and pUT18C_*rv0613c*_314–550_ (right plate); (**B**) Red colony phenotype observed on MacConkey agar plates for *E. coli* transformed with pKT25_*hbhA* and pUT18C_*mmpL14*_519–749_ (middle plate) in contrast to the *E. coli* transformed with pKT25_*hbhA* and pUT18C (left plate) or pKT25 and pUT18C_*mmpL14*_519–749_ (right plate); (**C**) Red colony phenotype observed on MacConkey agar plates for *E. coli* transformed with pKT25_*hbhA*_1–109_ and pUT18C_*rv0613c*_314–550_ or pUT18C_*mmpL14*_519–749_ (right plates) in contrast to the *E. coli* transformed with pKT25_*hbhA*_1–109_ and pUT18C (left plates).

To identify protein partners able to interact with HBHA, we used the Bacterial Adenylate Cyclase Two-Hybrid (BACTH) system with pKT25_*hbhA* as a bait to screen a *Mtb* Erdman library contained in pUT18C [11]. The initial bacterial colonies selected based on their red color on MacConkey agar were further transferred onto fresh selective plates to confirm the phenotype. DNA was then extracted from the selected colonies and the pUT18C inserts were sequenced. This led to the identification of a portion of the *rv0613c* and *mmpL14* genes. These portions code for amino acids 314 to 550 and 519 to 749 of Rv0613c and MmpL14, respectively. The *rv0613c* gene from *Mtb* Erdman is 100% identical to that of *Mtb* H37Rv. The *mmpL14* gene is absent from *Mtb* H37Rv but completely present in *Mtb* Erdman [12,13]. pUT18C containing the truncated *rv0613c*_314–550_ or *mmpL14*_519–749_ were re-introduced into *Escherichia coli* DHM1 containing pKT25_*hbhA* to confirm the phenotype in the BACTH system. For the bacteria expressing *rv0613c*_314–550_, all the colonies displayed a red color on MacConkey agar plates, whereas for the bacteria expressing *mmpL14*_519–749_, the red labeling was more heterogeneous (Figure 1A). As expected, *E. coli* DHM1 containing pKT25_*hbhA* together with empty pUT18C did not yield any red colonies, nor did *E. coli* DHM1 containing empty pKT25 together with pUT18C_*rv0613c*_314–550_ or pUT18C_*mmpL14*_519–749_ (Figure 1A).

The C-terminal part of HBHA contains the heparin-binding domain (HBD) [14], a 39 amino acid region enriched in lysine, alanine and proline residues, which could potentially lead to non-specific interactions with other proteins. In order to determine whether this C-terminal domain of HBHA is responsible for its interactions with Rv0613 and/or MmpL14, we introduced the partial *hbhA* DNA fragments coding for HBHA_1–109_, devoid of its linker and HBD regions into pKT25 [15]. When this construct was used as a bait in the BACTH system, co-expressed with either *rv0613c*_314–550_ or *mmpL14*_519–749_, it yielded red colonies in both cases, again more consistently with *rv0613c*_314–550_ than with *mmpL14*_519–749_ (Figure 1B). These results indicate that the N-terminal moiety of HBHA interacts with Rv0613 and MmpL14. In addition, as only the C-terminal part of HBHA is methylated, this implies that methylation does not seem to be important for HBHA interactions with Rv0613c or MmpL14. All these interactions were further confirmed by using LB agar plates supplemented with IPTG and X-gal (Supplementary Figure S1).

### 2.2. Rv0613c Is Conserved within the Mtb Complex and Contains a SEC-C Motif

**Figure 2:**
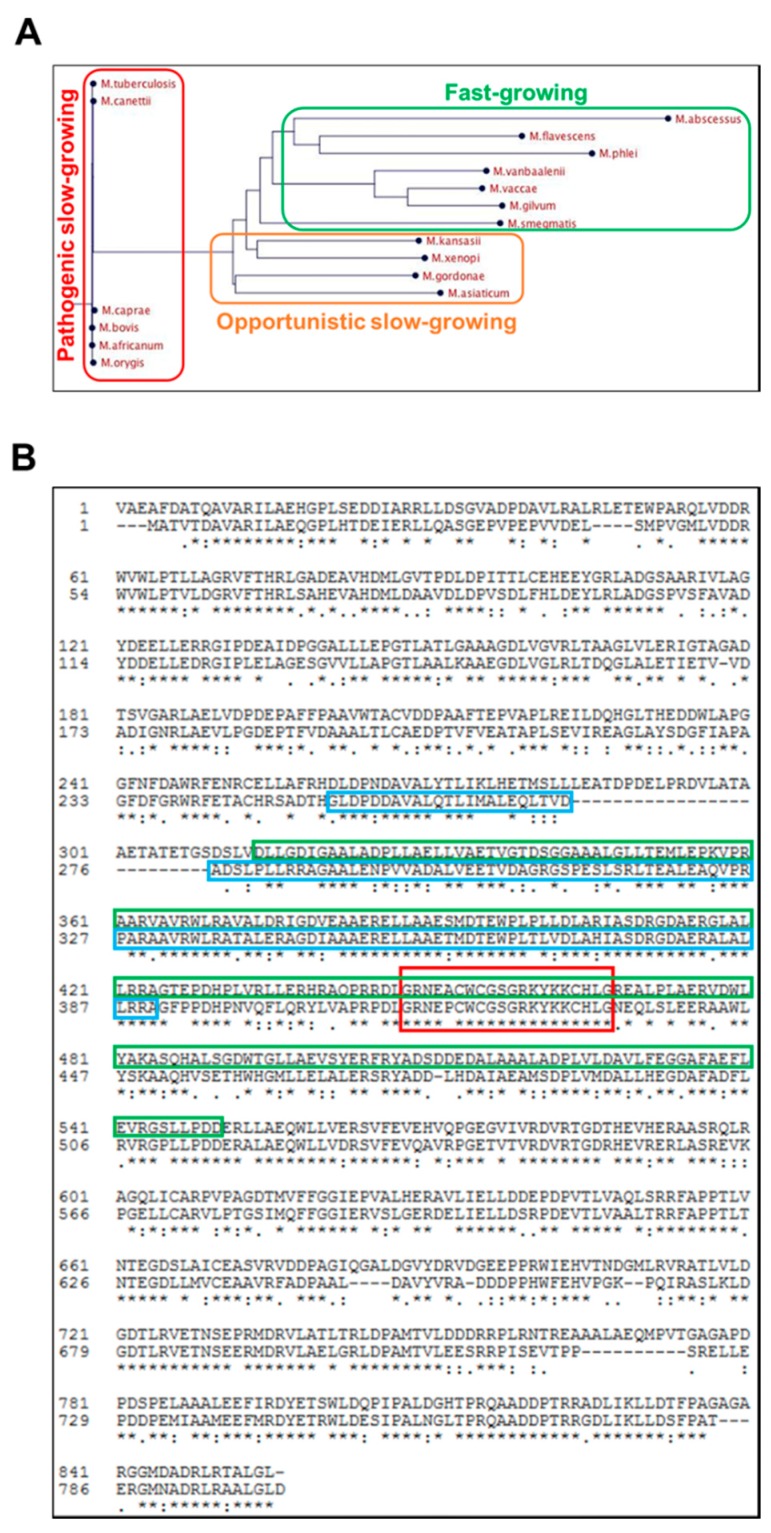
MSMEG_1285 is an orthologue of Rv0613c. (**A**) Phylogenetic tree representing Rv0613c homologues among different mycobacteria; (**B**) Sequence alignment of Rv0613c (first line) and MSMEG_1285 (second line) using Clustal Omega. The red rectangle corresponds to the SEC-C motif. The region interacting with HBHA of Rv0613c_314–550_ is shown in the green rectangles, and the TPR of MSMEG_1285 is shown in the blue rectangles.

The *rv0613c* gene codes for a protein of 855 amino acids. Like HBHA, Rv0613c has orthologues only in other Actinobacteria and is strictly conserved among the *Mtb* complex (Figure 2A). Among fast-growing mycobacteria, the *Msmeg* protein *MSMEG_1285* is the closest orthologues of Rv0613c, displaying 59% sequence identity with full-length Rv0613c and 66% sequence identity with Rv0613c_314–550_ (Figure 2B). No orthologue was found in *M. leprae*, *M. marinum*, *M. ulcerans*, *M. avium*, *M. fortuitum* and *M. chelonae*.

In silico analyses of the Rv0613c sequence revealed poor folding predictions [16]. However, Rv0613c is annotated as a protein containing a SEC-C motif (around 20 amino acids), which is notably found in bacterial preprotein translocases. MSMEG_1285 also contains a SEC-C motif and an additional TetratricoPeptide Repeat (TPR) domain that may be involved in protein-protein interaction (Figure 2B). TPR-containing proteins are associated with various virulence mechanisms in several bacterial pathogens [17]. A homologous region of the TPR domain is present in almost its entire length in Rv0613c_314–550_ (Figure 2B). Therefore, this region may potentially be involved in the HBHA-Rv0613c interaction, although Rv0613c was not annotated as a protein containing such a domain, probably due to some sequence divergence between *Mtb* H37Rv and *Msmeg* mc^2^155 in the N-terminal part of the TPR domain.

### 2.3. Generation of a ΔMSMEG_1285 Mutant in Msmeg mc^2^155

To characterize the role of Rv0613c/MSMEG_1285, we used fast-growing *Msmeg* as a model system. A *MSMEG_1285* deletion mutant (Δ*MSMEG_1285*) was generated in *Msmeg* mc^2^155, as well as its complemented derivative (Supplementary Figure S2A,B), proving that *MSMEG_1285* is dispensable for *Msmeg* mc^2^155 growth in vitro. The absence of *MSMEG_1285* and the complementation were verified using two different sera from Rv0613c-immunized mice (Supplementary Figure S2C). Moreover, the total amount of intracellular HBHA_*Msmeg* was not impacted by the deletion of *MSMEG_1285* (Supplementary Figure S2D).

### 2.4. MSMEG_1285 Is Necessary for the Proper Cell-Surface Exposure of HBHA_Mtb

Atomic Force Microscopy (AFM) has been successfully used to examine the cell-surface exposure of HBHA in *Mycobacterium bovis* bacillus Calmette-Guérin (BCG) and its interaction with heparin coated on the AFM tip [18]. HBHA is a highly abundant protein, with up to 1% of total soluble proteins of BCG Pasteur. In contrast, HBHA_*Msmeg* is less abundant, explaining why the presence of an orthologous protein in *Msmeg* remained elusive for years [9]. In addition, the affinity between HBHA_*Msmeg* and heparin is much lower than the affinity between HBHA_*Mtb* and heparin [9]. Therefore, to monitor HBHA interaction with heparin, we transformed *Msmeg* mc^2^155 and the Δ*MSMEG_1285* with pMV361_*hbhA_EGFP*, allowing heterologous expression of *hbhA_Mtb*. The fusion with EGFP did not affect cell surface-exposure of HBHA in BCG Pasteur [19] and was further used to distinguish between endogenous HBHA_*Msmeg* and the recombinant HBHA_*Mtb*.

**Figure 3:**
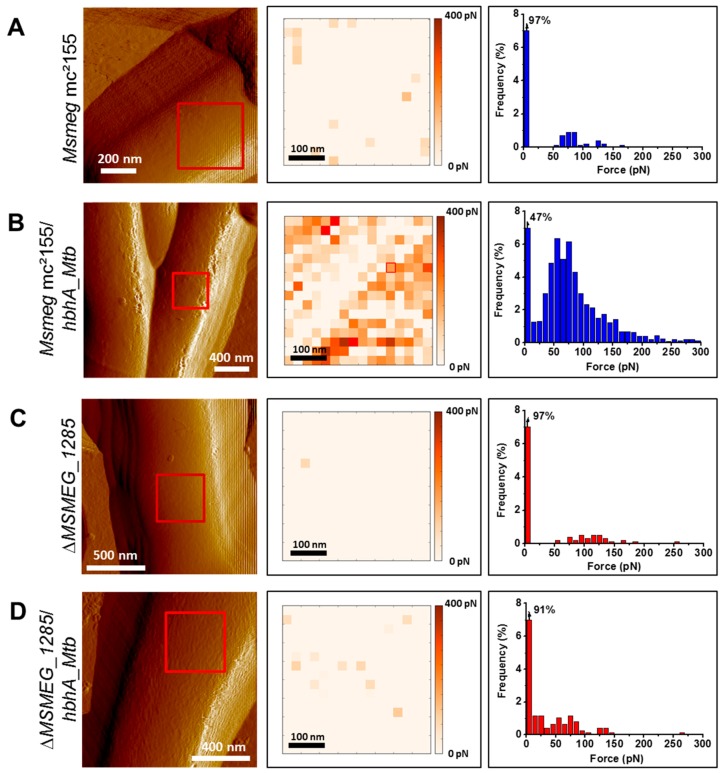
Deletion of *MSMEG_1285* in *Msmeg* mc^2^155 impacts the cell-surface exposure of HBHA_*Mtb*. AFM tomographic image (left panel), representative spatially-resolved map of adhesion forces recorded with AFM heparin-coated tip (medium panel) and corresponding histogram (right panel, obtained from 756 force curves) for (**A**) the wild-type strain of *Msmeg*, (**B**) *Msmeg* mc^2^155 expressing *hbhA_Mtb*, (**C**) the Δ*MSMEG_1285* mutant and (**D**) the Δ*MSMEG_1285* mutant expressing *hbhA_Mtb*. All the strains were cultured in 7H9 supplemented with OADC and without detergent. For each image, the red square corresponds to the area scanned for the determination of one adhesion map.

When grown in 7H9 plus OADC without detergent, the absence of MSMEG_1285 was found to significantly decrease the binding between heparin and HBHA_*Mtb* produced in *Msmeg*, as evidenced by the poor interaction of HBHA_*Mtb* produced in the Δ*MSMEG_1285* mutant with the AFM heparin-coated tip. Only 47% of the measurements did not detect adhesion events in recombinant *Msmeg* mc^2^155 producing HBHA_*Mtb* (Figure 3B), whereas this increased to 91% of measurements in the Δ*MSMEG_1285* mutant (Figure 3D). Without recombinant production of HBHA_*Mtb*, the wild-type strain or the Δ*MSMEG_1285* mutant barely interacted with the AFM heparin-coated tip as 97% of the measurements did not detect adhesion events in either case (Figure 3A,C), confirming that HBHA_*Msmeg* has low affinity for heparin and therefore does not interfere with AFM measurement. When grown in Sauton medium (supplemented with soft detergent 0.025% tyloxapol), the difference between the two recombinant strains producing *hbhA*_*Mtb* was confirmed, with 24% and 79% of measurements not detecting any adhesion events for *Msmeg* mc^2^155 and the Δ*MSMEG_1285* mutant, respectively (Supplementary Figure S3B,D). Here again, recombinant production of HBHA_*Mtb* was necessary, as 97% of the measurements with the wild-type strain did not show any adhesion events (Supplementary Figure S3A) while 81% of the measurements with the Δ*MSMEG_1285* mutant did not give any adhesion events (Supplementary Figure S3C). These results thus demonstrate that MSMEG_1285 is necessary for the proper cell-surface exposure of HBHA_*Mtb* in *Msmeg* mc^2^155.

### 2.5. Loss of Auto-Aggregation in ΔMSMEG_1285 Expressing hbhA_Mtb

**Figure 4:**
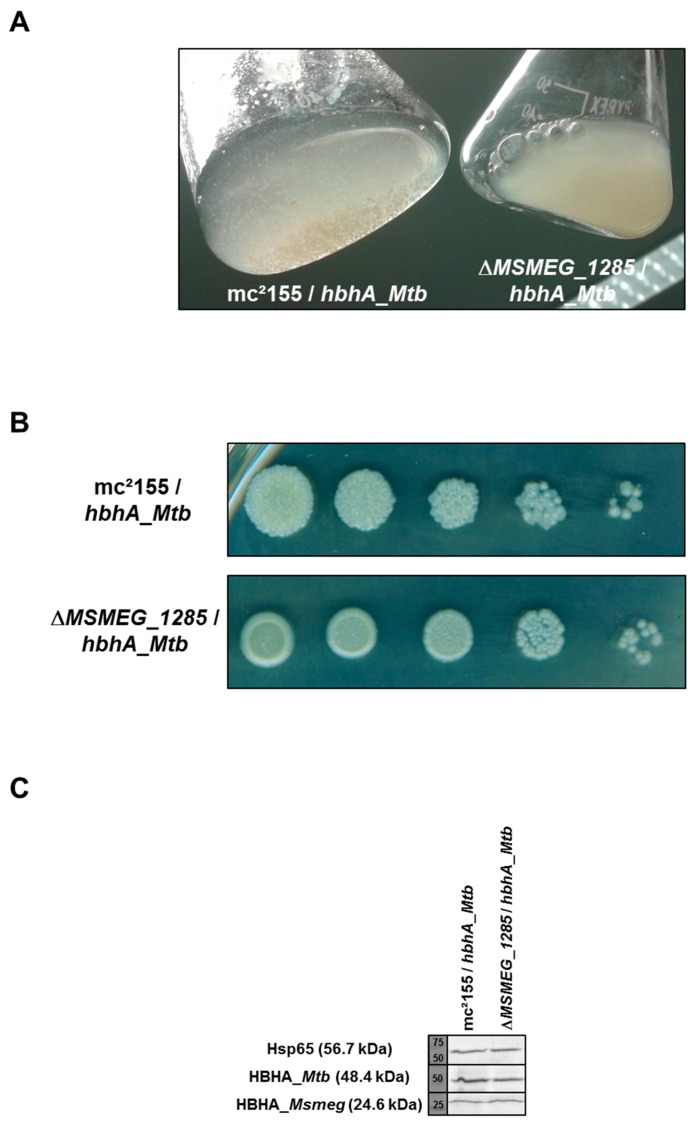
Heterologous expression of *hbhA_Mtb* in the Δ*MSMEG_1285* mutant affects auto-aggregation and colony morphology. (**A**) Overnight cultures in 7H9 supplemented with OADC and without detergent of *Msmeg* mc^2^155 and the Δ*MSMEG_1285* mutant, both expressing *hbhA_Mtb*; (**B**) Serial dilution of *Msmeg* mc^2^155 (upper series) and the Δ*MSMEG_1285* mutant (lower series), both expressing *hbhA_Mtb*, on 7H11 agar plates; (**C**) Western-blot analysis using monoclonal antibodies anti-Hsp65 (upper panel), anti-HBHA VF2 (medium panel) and anti-HBHA D2 (lower panel) on 40 µg of total lysates from *Msmeg* mc^2^155 and the Δ*MSMEG_1285* mutant, both expressing *hbhA_Mtb*.

Surface-exposed HBHA_*Mtb* has been shown to cause auto-aggregation of mycobacteria [2]. To assess the role of MSMEG_1285 in auto-aggregation, recombinant *Msmeg* mc^2^155 and Δ*MSMEG_1285* producing HBHA_*Mtb* were grown in 7H9 medium supplemented with OADC in the absence of detergent. Production of recombinant HBHA_*Mtb* in *Msmeg* mc^2^155 induced clumping of the mycobacteria, consistent with previous work [2] (Figure 4A). In contrast, in the same culture conditions, the Δ*MSMEG_1285* mutant producing HBHA_*Mtb* failed to form clumps (Figure 4A). This phenotype was related to the recombinant production of HBHA_*Mtb* as the non-recombinant strains did not display any difference in liquid cultures (Supplementary Figure S4A). When grown on 7H11 agar plates, the colonies of the Δ*MSMEG_1285* mutant expressing *hbhA*_*Mtb* displayed a smooth and round phenotype, whereas *Msmeg* mc^2^155 expressing *hbhA*_*Mtb* displayed a rough phenotype (Figure 4B). Again, this phenotype was related to the recombinant production of HBHA_*Mtb* as the non-recombinant strains grew as rough colonies on 7H11 agar plates (Supplementary Figure S4B). As the level of recombinant HBHA_*Mtb* in fusion with EGFP (48.4 kDa for hybrid protein) or endogenous HBHA_*Msmeg* (24.6 kDa) production by *Msmeg* was not impacted by the Δ*MSMEG_1285* deletion (Figure 4C), this infers that the clumping and the rough phenotypes are mediated by a MSMEG_1285-dependent surface exposure of HBHA_*Mtb*.

### 2.6. MSMEG_1285 Is Necessary for Full Infectivity of Msmeg mc^2^155 Expressing hbhA_Mtb

**Figure 5:**
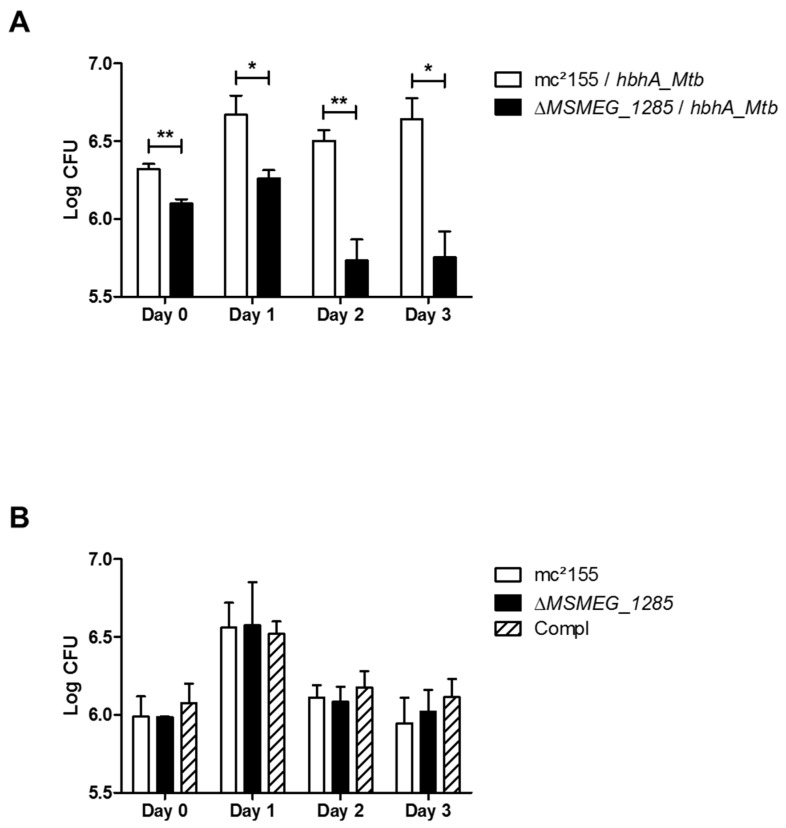
Heterologous expression of *hbhA_Mtb* in the Δ*MSMEG_1285* mutant impairs its infectivity towards A549 cells (**A**) Mean values of CFU counting for the recombinant *M. smegmatis* mc^2^155 (white bars) and Δ*MSMEG_1285* mutant (black bars), both expressing *hbhA_Mtb*, after infection of A549 cells; (**B**) CFU counting for the non-recombinant *M. smegmatis* mc^2^155 (white bars), Δ*MSMEG_1285* mutant (black bars) and complemented strain (striped bars), after infection of A549 cells. The two graphs are representative of two independent experiments. *, *p* < 0.05; **, *p* < 0.01.

It has previously been shown that HBHA_*Mtb* mediates the interaction between *Mtb* and epithelial cells, leading to extrapulmonary dissemination of the bacilli [5]. Using the epithelial cell line A549, we examined the impact of *MSMEG_1285* deletion on HBHA_*Mtb*-mediated cytoadherence. Therefore, *Msmeg* mc^2^155, Δ*MSMEG_1285* and their recombinant HBHA_*Mtb*-producing derivatives were used to infect A549 cells. A difference was observed after 4 h of infection (day 0), as a higher number of colony-forming units (CFU) were detected on cells incubated with recombinant *Msmeg* mc^2^155 expressing *hbhA_Mtb* compared to the recombinant Δ*MSMEG_1285* mutant (Figure 5A). This difference was maintained after three days after of incubation (Figure 5A). This phenotype was dependent on HBHA_*Mtb* as the non-recombinant Δ*MSMEG_1285* mutant did not differ from non-recombinant *Msmeg* mc^2^155 or the complemented strain in its ability to infect A549 infectivity from day 0 to day 3 (Figure 5B), indicating that MSMEG_1285 is required for HBHA_*Mtb*-mediated adherence to epithelial cells.

### 2.7. Proteomic Analysis of the ΔMSMEG_1285 Mutant

In order to determine whether any protein is affected in its localization by the deletion of *MSMEG_1285*, the protein content of the membrane (MB) and cell wall (CW) fractions of *Msmeg* mc^2^155, the *MSMEG_1285* mutant and the complemented strains, as well as the recombinant *Msmeg* strains expressing *hbhA_Mtb*, was examined by mass spectrometry analyses. Using a spectral counting approach with a ratio ≤0.2 for the total number of spectra (mutant/wild type and mutant/complemented strain), no protein was lacking in the MB fraction of the Δ*MSMEG_1285* mutant when compared with either the wild type or the complemented strain. However, surprisingly, using a spectral counting approach with a ratio ≥5, one protein, MSMEG_3496, appeared to be enriched in the MB fraction of the Δ*MSMEG_1285* mutant in comparison with both the wild type and complemented strains (Table 1). Fourteen spectra for MSMEG_3496 were detected in the mutant, whereas only one spectrum was detected for recombinant *Msmeg* mc^2^155 (Table 1). Thus, the absence of MSMEG_1285 led to an enrichment of MSMEG_3496 in the *Msmeg* MB fraction. However, this tendency was not confirmed in the CW fraction of recombinant strains (Table 1).

The total number of spectra for HBHA_*Mtb* was similar between recombinant *Msmeg* mc^2^155 and Δ*MSMEG_1285* mutant (Table 1), confirming that the phenotypes observed above were not due to differences in protein content of the MB or CW fractions, but were due to differences in HBHA cell-surface exposure between *Msmeg* mc^2^155 and the MSMEG_1285-deficient mutant. As expected, MSMEG_1285 was not detected in the Δ*MSMEG_1285* mutant.

## 3. Discussion

Considering the importance of HBHA in *Mtb* pathogenesis [5], unraveling its biosynthesis and its function is a crucial step in understanding the ability of the bacilli to interact with epithelial cells and to undergo extrapulmonary dissemination. HBHA was previously detected at the cell-surface of the bacilli [2], although it lacks a canonical signal peptide for transport. Here, we describe Rv0613c and MmpL14 as the first proteins identified to interact with HBHA. Using the BACTH system, the HBHA interaction with Rv0613c was consistently strong, whereas the interaction was less consistent with MmpL14, as the red phenotype displayed by *E. coli* cells on McConkey agar plates in the BACTH system appeared to be more heterogeneous, in comparison with the HBHA-Rv0613c_314–550_ interaction. However, this heterogeneity could be the consequence of using a truncated version of MmpL14 (i.e., MmpL14_519–749_) in BACTH system, and other portions of MmpL14 may be needed to retain full interaction with HBHA. Therefore, we focused this study on Rv0613c, for which we could identify its homolog in *Msmeg* as MSMEG_1285.

HBHA has several functional domains, the best studied of which is the C-terminal domain, containing several lysine-proline-alanine-rich repeats. This domain has been shown to be involved in mycobacterial binding to epithelial cells [2,20]. The BACTH studies described here show that it is not involved in binding to Rv0613c, since BACTH experiments done with a truncated version of HBHA, lacking this C-terminal domain, gave the same results as those obtained with full-length HBHA (Figure 1A,B). This implies that the HBHA-Rv0613c interaction is not based merely on ionic interactions of the positively charged C-terminal domain of HBHA with Rv0613c, which could lack specificity. It rather suggests a more specific protein-protein interaction. As HBHA is an intrinsically disordered protein [15], it may therefore be possible that its interaction with Rv0613c induces a specific conformation of HBHA enabling it to be properly exposed and oriented at the bacterial surface and to carry out its biological function as an adhesin and a bacterial clumping factor.

Both Rv0613c and MSMEG_1285 contain a SEC-C motif (Figure 2B), notably found in bacterial preprotein translocases. It is therefore tempting to speculate that Rv0613c and MSMEG_1285 fulfill a role similar to that of the preprotein translocase SecA and the chaperone SecB in order to facilitate HBHA export through the mycobacterial cytoplasmic membrane and HBHA cell surface exposure. Accordingly, the deletion of *MSMEG_1285* led to the impairment of the proper cell surface exposure of recombinant HBHA_*Mtb* produced in *Msmeg*, as evidenced by AFM (Figure 3, Supplementary Figure S3). The deletion also led to a decrease in mycobacterial auto-aggregation by the HBHA_*Mtb*-producing *Msmeg* strain (Figure 4A), to a smooth colony phenotype when grown on Middlebrook 7H11 agar plates (Figure 4B) and to a decrease in infectivity of A549 cells (Figure 5A). Native HBHA has also been reported to form homodimers, and it was previously suggested that the HBHA trans-dimerization is responsible for bacterial agglutination [21]. Therefore, a defect in HBHA cell surface exposure and orientation would directly lead to the loss of the auto-aggregation, to a smooth phenotype, and a decrease in infectivity. Importantly, the formation of large mycobacterial aggregates has been reported to increase cytotoxicicty and to enhance effective killing of host cells after macrophage internalization, highlighting the importance of aggregation state in *Mtb* virulence [22].

Although Rv0613c has no significant predicted structural similarities with hydrolases or peptidases, it was recently shown to display protease activity, as evidenced by its ability to cleave β–casein [16]. It was suggested that either Rv0613c is a divergent protease or that its protease activity is a moonlighting function [16]. However, we have no evidence that Rv0613c exerts a protease activity on HBHA. Interestingly, the proteases tested by Ortega et al. [16], Rv0525 and Rv1192, with a molecular weight of 22 and 30 kDa, respectively, are three- to four-fold smaller than the 93-kDa Rv0613c, suggesting that Rv0613c may harbor several subdomains with different activities. In the BACTH assay, the portion of Rv0613c (Rv0613c_314–550_) interacting with HBHA represents only one quarter of the entire Rv0613c protein. Thus, the protease subdomain of Rv0613c could be distinct from the HBHA-interacting subdomain. It remains to be determined whether the protease activity of Rv0613c plays any role in HBHA maturation process.

In addition to the SEC-C motif, MSMEG_1285 also contains a TPR domain. In spite of some divergence in the N-terminal part, the C-terminal part of the MSMEG_1285 TPR domain is very similar to the corresponding sequence of Rv0613c (Figure 2B), strongly suggesting that Rv0613c also contains a TPR domain, although it was originally not annotated as such in protein databases. However, the web-based TPRpred tool (Available online: https://toolkit.tuebingen.mpg.de/#/tools/tprpred) predicts that _364_VAVRW…MDTEW_397_ and _398_PLPLL…EPDHP_431_ of Rv0613c are TPR peptides with a *p*-value of 1.6 × 10^-5^ and 4.1 × 10^-4^, respectively. This portion of Rv0613c is also present in the fragment identified by the BACTH system to interact with HBHA. TPR domains are known to mediate protein-protein interactions and TPR-containing proteins are often involved in the biogenesis of bacterial secreted or surface proteins [17]. For example, the assembly machinery of the β-barrel outer membrane protein of *E. coli* consists of a complex of five proteins, one of which, named BamD, contains 5 TPR domains [23]. This protein binds to unfolded outer membrane proteins prior to folding and insertion into the outer membrane [24,25]. ClustalO predicts 21% of sequence identity between the TPR1 of BamD and Rv0613c_471–546_ (Supplementary Figure S5), which is contained within the portion of Rv0613c that interacts with HBHA in the BACTH system. Similarly, the TPR-containing protein TprA from *Porphyromonas gingivalis* is also involved in the outer-membrane display of the TapA protein, most likely via protein-protein interaction involving the TPR domain of TprA and this mechanism is required to maintain full infectivity of epithelial cells by *P. gingivalis* [26]. Thus, it is tempting to speculate that a similar mechanism, involving protein-protein interactions with the TPR domain of Rv0613c, may be at play to address HBHA to the mycobacterial cell surface in its proper orientation. Interestingly, both SEC-C motif- and TPR domain-containing proteins are enriched in the pathogenic *Mtb* clade in comparison with non-pathogenic mycobacteria [27], which suggests their involvement in virulence traits.

It was of interest to investigate whether the sub-cellular localization of proteins was also affected by the deletion of MSMEG_1285. Surprisingly, an overrepresentation (cut-off ≥ 5) of a single *Msmeg* protein, MSMEG_3496, was seen in the MB fraction of the mutant as compared to *Msmeg* mc^2^155. This protein is one of the orthologues of MmpL5 (Rv0676c) in *Mtb*. It is intriguing that the second protein, MmpL14, found here to interact with HBHA in the BACTH system is also a member of the MmpL family. The *mmpL14* gene is part of the RvD2 deleted region and is absent in *Mtb* H37Rv [12,13], but conserved among the majority of *Mtb* strains, including CDC1551 (locus MT1802) and H37Ra [28,29], as well as most of the sequenced *Mtb* clinical isolates. The MmpL protein family has been shown to be involved in substrate transport, such as the transport of lipids through the mycobacterial membrane, siderophore export and iron acquisition, and drug efflux [30,31]. MmpL14_519–749_ comprises the complete docking subdomain DC as well as part of the porter subdomains PC1 and PC2, making this part of the protein accessible for interactions with surface-exposed proteins. The periplasmic docking subdomain DC was suggested to play a role in protein-protein interactions [32], consistent with our findings on its interaction with HBHA. Moreover, the rough to smooth transition phenotype cannot be reduced only to the impairment of HbhA transport. Interestingly, Bernut and coworkers reported that a Y842H mutation in *M. bolletii* MmpL4a was involved in the smooth to rough transition phenotype [33]. The authors proposed that this single key mutation has an impact of the 3D structure of the protein, affecting the proton motive force. Considering the interaction of HBHA with MmpL14, the rough to smooth transition phenotype could be an indirect consequence of the improper HBHA cell surface exposure, also compromising the structural organization and/or exposure of MmpL proteins. In addition, as MmpL proteins are key players in the transport of lipids, the authors suggested that a functional link may exist between MmpL4a and the GPL biosynthesis [33]. We can therefore not exclude link between HBHA and the MmpL proteins during the biogenesis or function of HBHA. Future work will address this issue.

## 4. Materials and Methods

### 4.1. Bacterial Strains and Media

*E. coli* TOP10 (Invitrogen, Carlsbad, CA, USA) was used for cloning and *E. coli* DHM1 was used for BACTH assays (Table S1). The *E. coli* strains were grown in LB medium (MP Biomedicals, Illkirch, France) at 37 °C supplemented with ampicillin (100 μg·mL^−1^), hygromycin (50 μg·mL^−1^) or kanamycin (25 μg·mL^−1^), when required. *Msmeg* mc^2^155, the deletion mutant Δ*MSMEG_1285* and the corresponding complemented strain (Table S1) were grown in Sauton’s medium (containing 0.025% tyloxapol), in Middlebrook 7H9 broth or on 7H11 agar plates supplemented with OADC enrichment (Becton Dickinson, Le Pont-de-Claix, France), hygromycin (50 μg·mL^−1^) or kanamycin (25 μg·mL^−1^), when required.

### 4.2. Generation of the ΔMSMEG_1285 Mutant in Msmeg mc^2^155

To generate the Δ*MSMEG_1285* mutant, we followed the protocol previously described by van Kessel et al. [34]. Briefly, upstream and downstream regions of *MSMEG_1285* were amplified from *Msmeg* mc^2^155 genomic DNA using primers [Up_*MSMEG1285*_dir and Up_*MSMEG1285*_rev] and [Down_*MSMEG1285*_dir and Down_*MSMEG1285*_rev], respectively (Table S2). These upstream and downstream fragments were inserted at either side of a hygromycin-resistance cassette in pJSC347 (Table S1). After digestion by SpeI and StuI, the allelic exchange substrate was electroporated into competent *Msmeg* mc^2^155 containing pJV53 (Table S1), following induction by 0.2% acetamide for 24 h. Several hygromycin-resistant clones were selected and tested for the insertion of the hygromycin-resistance cassette by PCR using primers Seq_*MSMEG1285*_1, Seq_*MSMEG1285*_2 and Seq_*MSMEG1285*_3 (Supplementary Figure S2A,B and Table S2). The resulting PCR fragments were further checked by sequencing to confirm the insertion at the expected *Msmeg* mc^2^155 locus.

### 4.3. Plasmid Constructions

Different portions of *hbhA* were amplified by PCR using *Mtb*H37Rv genomic DNA and the pair of primers [pKT25_*hbhA*_dir and pKT25_*hbhA*_rev] or [pKT25_*hbhA*_dir and pKT25_*hbhA*_1–109__rev] (Table S2). The PCR fragments were digested with BamHI and KpnI and ligated into pKT25 digested with the same enzymes, thereby generating pKT25_*hbhA* and pKT25_*hbhA*_1–109_ (Table S1). The *MSMEG_1285* gene was amplified by PCR using *Msmeg* mc^2^155 genomic DNA and the pair of primers [pVV16_*MSMEG1285*_dir and pVV16_*MSMEG1285*_rev] (Table S2). The PCR fragment was digested with NdeI and HindIII and ligated into pVV16 digested with the same enzymes, thereby generating pVV16_*MSMEG_1285* (Table S1). All plasmids were checked by sequencing.

### 4.4. Bacterial Adenylate Cyclase Two-Hybrid Assays

One hundred ng of the *Mtb* Erdman library contained in pUT18C was electroporated into *E. coli* DHM1 containing pKT25_*hbhA*. Transformants were spread onto MacConkey agar (Becton Dickinson) plates supplemented with 100 μg·mL^−1^ ampicillin, 25 μg·mL^−1^ kanamycin, 0.5 mM IPTG and 1% maltose. After 4 to 6 days at 30 °C, clones were selected based on their red color and transferred onto fresh MacConkey agar plates to confirm the red phenotype. DNA was extracted from the selected colonies and pUT18C inserts were sequenced using the Seq_pUT18C_dir primer (Table S2). To confirm the phenotype, pUT18C containing *rv0613c*_314–550_ was re-introduced into *E. coli* DHM1 containing either pKT25_*hbhA* or pKT25_*hbhA*_1–109_. Transformants were spread onto both MacConkey agar plates and LB agar plates supplemented with 100 μg·mL^−1^ ampicillin, 25 μg·mL^−1^ kanamycin, 0.5 mM IPTG and 50 µg·mL^−1^ X-gal and incubated for 3 days at 30 °C.

### 4.5. Fractionation Protocol

*Msmeg* strains were disrupted in a French pressure cell and the lysates were centrifuged at 3000× *g* for 10 min at 4 °C to eliminate cellular debris and non-lysed cells. The supernatants were pelleted at 27,000× *g* for 30 min at 4 °C in order to collect cell-wall fractions (CW). The remaining supernatants were centrifuged at 100,000× *g* for 2 h at 4 °C to separate the membrane fractions (MB) from cytosolic fractions. Each fraction was resuspended in an appropriate volume of Laemmli buffer.

### 4.6. Immunoblot Analysis

Proteins were resolved by SDS-PAGE using 12% acrylamide gels and then electro-transferred onto a nitrocellulose membrane. The membrane was saturated with Tris-Buffered Saline (pH 7.5, TBS) containing 0.05% Tween 80 and 5% milk and probed overnight at 4 °C with anti-HBHA monoclonal antibody VF2 or D2 diluted 1:100 in TBS-Tween-3% milk or sera from Rv0613c-immunized mice diluted 1:100 in TBS-Tween-3% milk or anti-Hsp65 monoclonal antibody (Abcam, Cambridge, UK) diluted 1:1000 in TBS-Tween-3% milk. Finally, the membrane was incubated for 1 h at room temperature with goat anti-mouse horseradish peroxidase-conjugated secondary antibodies (Abcam), diluted 1:5000 in TBS-Tween-3% milk. Detection was performed using the Amersham ECL Prime Western-Blotting Detection Reagent (Fisher Scientific, Illkirch, France) and chemiluminescence was detected using the Amersham Imager 600 (GE Healthcare, Uppsala, Sweden).

### 4.7. Atomic Force Microscopy

Gold-coated cantilevers were cleaned with plasma oxygen, rinsed with ethanol, dried with a gentle nitrogen flow and immersed overnight at room temperature in a 25 µg·mL^−1^ solution of biotinylated bovine serum albumin (BBSA) (Sigma, Saint-Quentin-Fallavier, France) in PBS. Following rinsing with PBS, the BBSA surfaces were exposed to a 10 µg·mL^−1^ solution of streptavidin (Sigma) in PBS for 2 h followed by thorough rinsing with PBS. Finally, the BBSA/streptavidin surfaces were immersed for 2 h in PBS containing 10 µg·mL^−1^ biotinylated heparin (Sigma) and rinsed with PBS. The mycobacteria were immobilized on porous polycarbonate membranes with a 3 µm pore size (Merck Millipore, Darmstadt, Germany). After filtering a concentrated bacterial suspension, the membrane was transferred to a Petri dish containing the imaging solution and gently agitated to remove non-immobilized bacteria. Then, a small piece of the membrane (1 cm × 1 cm square) was cut and the bottom side was quickly dried using precision wipes before being attached to a steel sample puck using a small piece of double side adhesive tape. The mounted samples were transferred into the AFM liquid cell while avoiding dewetting. AFM contact mode images and force-distance curves were obtained using a MultiMode 8 AFM (Bruker, Santa Barbara, CA, USA). Measurements were performed in PBS at room temperature using gold-coated Silicon Nitride cantilevers (OMCL-TR400PB, Olympus Ltd., Tokyo, Japan). The spring constants of the cantilevers were measured using the thermal noise method (NanoScope Analysis software version 8.15, Bruker), yielding values ranging from 0.022 to 0.026 N·m^−1^. All force measurements were performed using a constant approach and retraction speed of 1500 nm·s^−1^, and with an interaction time of 500 ms. Adhesion maps were obtained by recording 16 × 16 force curves on 400 nm × 400 nm areas of the cells, calculating the adhesion force values and displaying them as grey pixels. Histograms were obtained by pooling the data from several adhesion maps. Data analysis was done using in-house developed pyAF (python Atomic Force) software, version 1.5.1. All experiments were performed at least on four different bacteria and with two different heparin-coated AFM tips.

### 4.8. Infection of A549 Cells

One day prior to infection, 2 × 10^5^ A549 cells per well resuspended in DMEM containing 10% Fetal Calf Serum (FCS) were seeded in 24-well plates and incubated overnight at 37 °C under 5% CO_2_. Cultures of *Msmeg* mc^2^155, Δ*MSMEG_1285* and their recombinant HBHA_*Mtb*-producing derivatives were grown overnight at 37 °C with shaking. Bacteria were washed twice in PBS, resuspended in DMEM containing 10% FCS, treated for 15 min in a sonication bath and centrifuged for 2 min at 120× *g* to eliminate remaining clumps. After two washes with PBS, A549 cells were infected with bacterial suspensions at an MOI of 50 and further incubated for 4 h at 37 °C under 5% CO_2_. After 4 h, the cells were extensively washed 5 times with PBS and either resuspended in DMEM containing 10% FCS or lysed with 250 µL of 0.1% Triton X-100. Serial dilutions of the lysates were plated onto 7H11 agar plates and CFU were counted after 3 days of incubation at 37 °C. At each time point, the remaining cells were washed 5 times in PBS and resuspended in fresh DMEM containing 10% FCS or, alternatively, lysed and CFU counts determined similarly. Each measure was done in triplicate and each experiment was done independently at least twice.

### 4.9. Mass Spectrometry Proteomic Analysis

After heating at 100 °C in 5% SDS, 5% β-mercaptoethanol, 1 mM EDTA, 10% glycerol, 10 mM Tris-HCl (pH 8) for 3 min, protein samples were fractionated by SDS-PAGE on a 10% acrylamide gel. The electrophoretic migration was stopped as soon as the protein sample entered 0.3 cm into the separating gel. The gel was briefly stained with Coomassie Blue, and one band, containing the entire sample, was cut. In-gel digestion of gel slices was performed as previously described [35]. The UltiMate 3000 RSLCnano System (Fisher Scientific) was used for the separation of the protein digests. Peptides were automatically fractionated onto a commercial C18 reversed phase column (75 µm × 250 mm, 2 µm particle, PepMap100 RSLC column, Thermo Fisher Scientific, at 35 °C). Trapping was performed during 4 min at 5 μL·min^−1^, with solvent A (98% H_2_O, 2% acetonitrile and 0.1% formic acid). The peptides were eluted using two solvents A (0.1% formic acid in water) and B (0.1% formic acid in acetonitrile) at a flow rate of 300 nL·min^−1^. Gradient separation was 3 min at 3% B, 110 min from 3% B to 20% B, 10 min from 20% B to 80% B and maintained for 15 min at 80% B. The column was equilibrated for 6 min with 3% buffer B prior to the next sample analysis. Eluted peptides from the C18 column were analyzed by Q-Exactive instruments (Fisher Scientific). The electrospray voltage was 1.9 kV, and the capillary temperature was 275 °C. Full MS scans were acquired in the Orbitrap mass analyzer over *m*/*z* 400–1200 range with a 70,000 (*m*/*z* 200) resolution. The target value was 3 × 10^6^. Fifteen most intense peaks with charge state between 2 and 5 were fragmented in the higher-energy collision-activated dissociation cell with normalized collision energy of 27%, and tandem mass spectrum was acquired in the Orbitrap mass analyzer with a 17,500 (*m*/*z* 200) resolution. The target value was 1 × 10^5^. The ion selection threshold was 5 × 10^4^ counts, and the maximum allowed ion accumulation times were 250 ms for full MS scans and 100 ms for tandem mass spectrum. Dynamic exclusion was set to 30 s.

### 4.10. Proteomic Data Analysis

Raw data collected during nanoLC-MS/MS analyses were processed and converted into a *.mgf peak list format with Proteome Discoverer 1.4 (Fisher Scientific). MS/MS data were analyzed using search engine Mascot (version 2.4.0, Matrix Science, London, UK) installed on a local server. Searches were performed with a tolerance on mass measurement of 0.2 Da for precursor and 0.2 Da for fragment ions, against a composite target-decoy database (17,848 total entries) built with a *Msmeg* UniProt database (strain ATCC 700084/mc (2)155, taxo 246196, October 2014, 8805 entries) fused with the sequences of HBHA_EGFP, recombinant trypsin and a list of classical contaminants (119 entries). Cysteine carbamidomethylation, methionine oxidation, protein N-terminal acetylation and cysteine propionamidation were searched as variable modifications. Up to one missed trypsin cleavage was allowed. For each sample, peptides were filtered out according to the cutoff set for protein hits with 1 or more peptides larger than 8 residues, ion score >25, identity score >6, corresponding to a 1% false positive rate.

### 4.11. Statistical Analysis

For infection of A549 cells by the *Msmeg* strains, the statistical significance (*p*-value) was tested with a two-tailed unpaired *t*-test (GraphPad Prism version 5.04, GraphPad Software, CA, USA) and *p*-values < 0.05 were considered as significant.

## Figures and Tables

**Table 1:** Total number of spectra (and unique peptides) for each protein in Membrane (MB) and Cell Wall (CW) fractions.

Protein	MW	Fraction	mc^2^155	Δ*MSMEG_1285*	Complemented	mc^2^155/*hbhA_Mtb*	Δ*MSMEG_1285*/*hbhA_Mtb*
MSMEG_3496	106210	MB	1 (1)	5 (4)	1 (1)	1 (1)	14 (12)
		CW	4 (3)	9 (6)	4 (3)	48 (22)	15 (10)
MSMEG_1285	88003	MB	1 (1)	0	4 (3)	3 (3)	0
		CW	1 (1)	0	11 (8)	2 (2)	0
MSMEG_0919 (HBHA_*Msmeg*)	24562	MB	28 (12)	37 (13)	27 (11)	13 (7)	23 (11)
		CW	15 (9)	16 (10)	16 (7)	7 (5)	13 (8)
Rv0475_EGFP (HBHA_*Mtb*)	48472	MB	0	0	0	17 (14)	14 (10)
		CW	0	0	0	4(4)	5 (4)

## Supplementary Materials

Supplementary materials can be found at http://www.mdpi.com/1422-0067/19/6/1673/s1.
