# Supplementary material for: Rv0613c/MSMEG_1285 Interacts with HBHA and Mediates Its Proper Cell-Surface Exposure in Mycobacteria
Source: Int J Mol Sci. 2018 Jun 5;19(6):1673. doi: 10.3390/ijms19061673 (PMC6032435; doi:10.3390/ijms19061673)
Supplement: Supplementary file 1 [file ijms-19-01673-s001.zip › ijms-300868-SI.pdf]

Supplementary Files

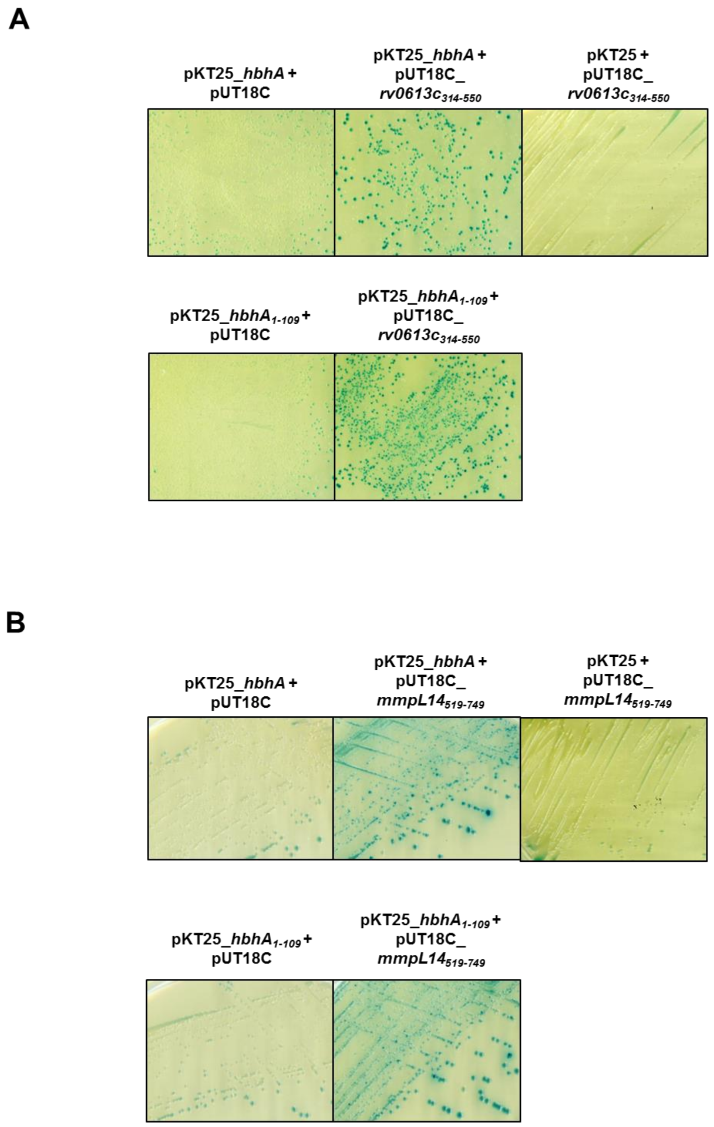

Supplementary Figure S1. HBHA interacts with Rv0613c and MmpL14 in the BACTH system. (**A**) Blue colony phenotype observed on LB agar plates supplemented with IPTG and X-gal for *E. coli* transformed with pKT25_*hbhA* and pUT18C_*rv0613c*$_{314-550}$ (upper panel, middle) and for *E. coli* transformed with pKT25_*hbhA*$_{1-109}$ and pUT18C_*rv0613c*$_{314-550}$ (lower panel, right) in contrast to the *E. coli* transformed with pKT25_*hbhA* and pUT18C (upper panel, left), *E. coli* transformed with pKT25_*hbhA*$_{1-109}$ and pUT18C (lower panel, left) and *E. coli* transformed with pKT25 and pUT18C_*rv0613c*$_{314-550}$ (upper panel, right); (**B**) Blue colony phenotype observed on LB agar plates supplemented with IPTG and X-gal for *E. coli* transformed with pKT25_*hbhA* and pUT18C_*mmpL14*$_{519-749}$ (upper panel, middle) and *E. coli* transformed with pKT25_*hbhA*$_{1-109}$ and pUT18C_*mmpL14*$_{519-749}$ (lower panel, right) in contrast to the *E. coli* transformed with pKT25_*hbhA* pUT18C (upper panel, left), *E. coli* transformed with pKT25_*hbhA*$_{1-109}$ and pUT18C (lower panel, left) and *E. coli* transformed with pKT25 and pUT18C_*mmpL14*$_{519-749}$ (upper panel, right).

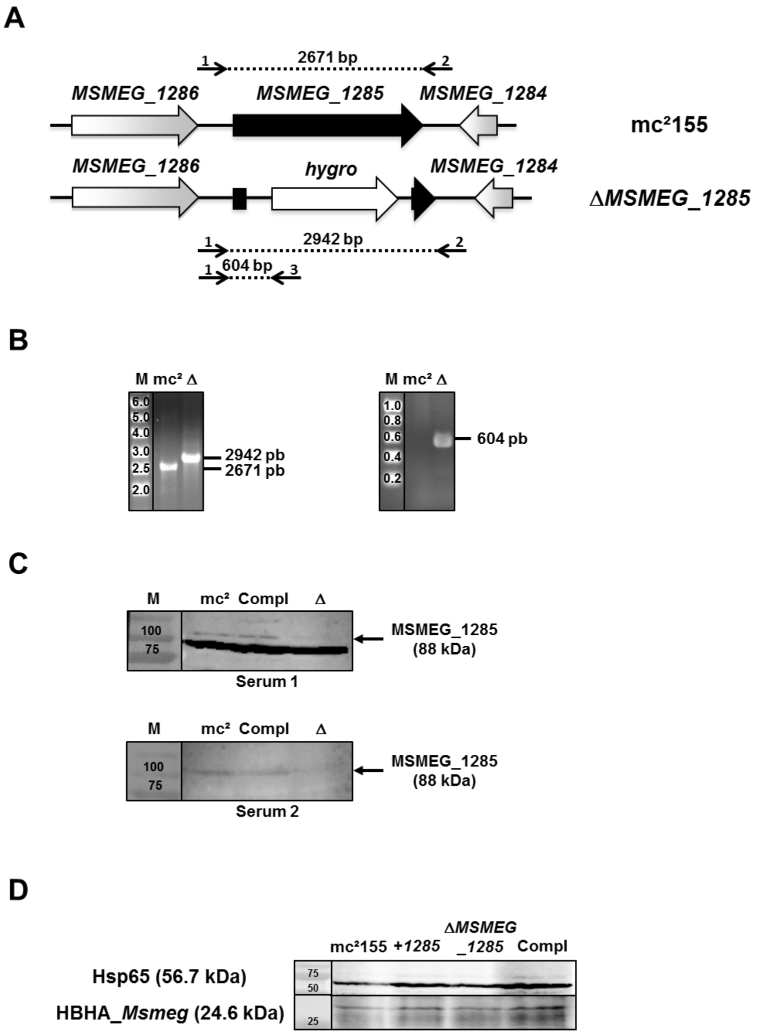

Supplementary Figure S2. Generation of the Δ*MSMEG_1285* mutant in *Msmeg* mc²155. (**A**) Genomic organization of the *MSMEG_1285* locus in *Msmeg* mc²155 and schematic representation for the insertion of the hygromycin resistance cassette. The large black arrows depict the *MSMEG_1285* gene (complete, upper line; interrupted, lower line). The grey arrows show the *MSMEG_1285* flanking genes, and the white arrow indicates the hygromycin resistance cassette. The small black arrows symbolize primers 1, 2 and 3 used for PCR verifications. Numbers in bp over the dotted lines indicate the expected base-pair numbers of the PCR fragments; (**B**) PCR verifications using the pairs of primers (1-2, left panel) and (1-3, right panel) with genomic DNA extracted from *Msmeg* mc²155 (mc²) and the Δ*MSMEG_1285* mutant (Δ). The expected sizes of the PCR amplicons are indicated in bp in the right margins, and the sizes of the size markers are given in the left columns, expressed in kbp; (**C**) Western-blot analysis using two different sera from Rv0613c-immunized mice on total lysates of *Msmeg* mc²155, the complemented strain (Compl) and the Δ*MSMEG_1285* mutant; (**D**) Western-blot analysis using anti-Hsp65 (upper panel) and anti-HBHA 5F2 (lower panel) monoclonal antibodies on total lysates of *Msmeg* mc²155, *Msmeg* mc²155 containing pVV16_*MSMEG_1285* (+*1285*), the Δ*MSMEG_1285* mutant and the complemented strain (Compl).

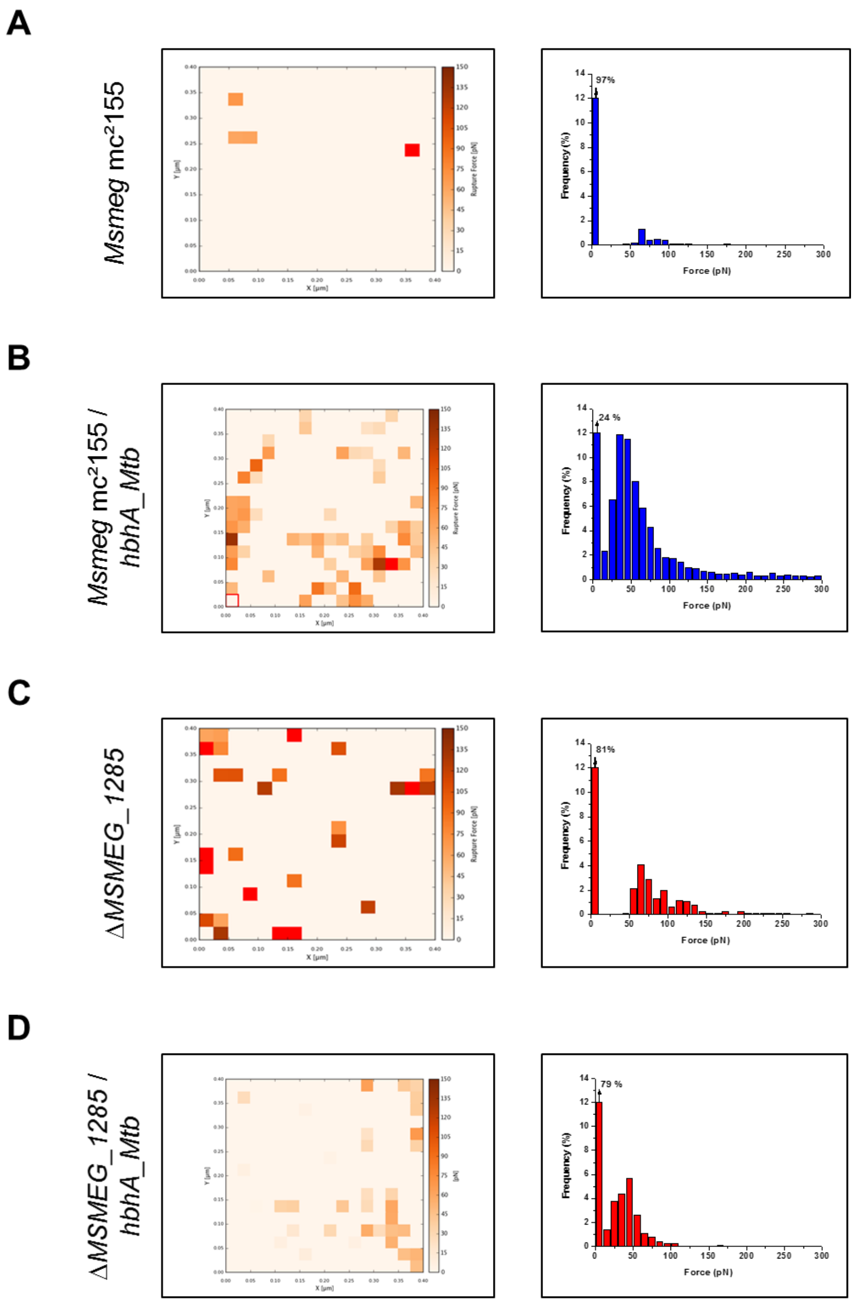

Supplementary Figure S3. Deletion of *MSMEG_1285* in *Msmeg* mc²155 impacts the cell-surface exposure of HBHA_*Mtb*. Representative spatially-resolved map of adhesion forces recorded with AFM heparin-coated tip (left panel) and corresponding histogram (right panel, obtained from 756 force curves) for (**A**) the wild-type strain of *Msmeg*, (**B**) *Msmeg* mc²155 expressing *hbhA_Mtb*, (**C**) the Δ*MSMEG_1285* mutant and (**D**) the Δ*MSMEG_1285* mutant expressing *hbhA_Mtb*. All the strains were cultured in Sauton with 0.025% tyloxapol.

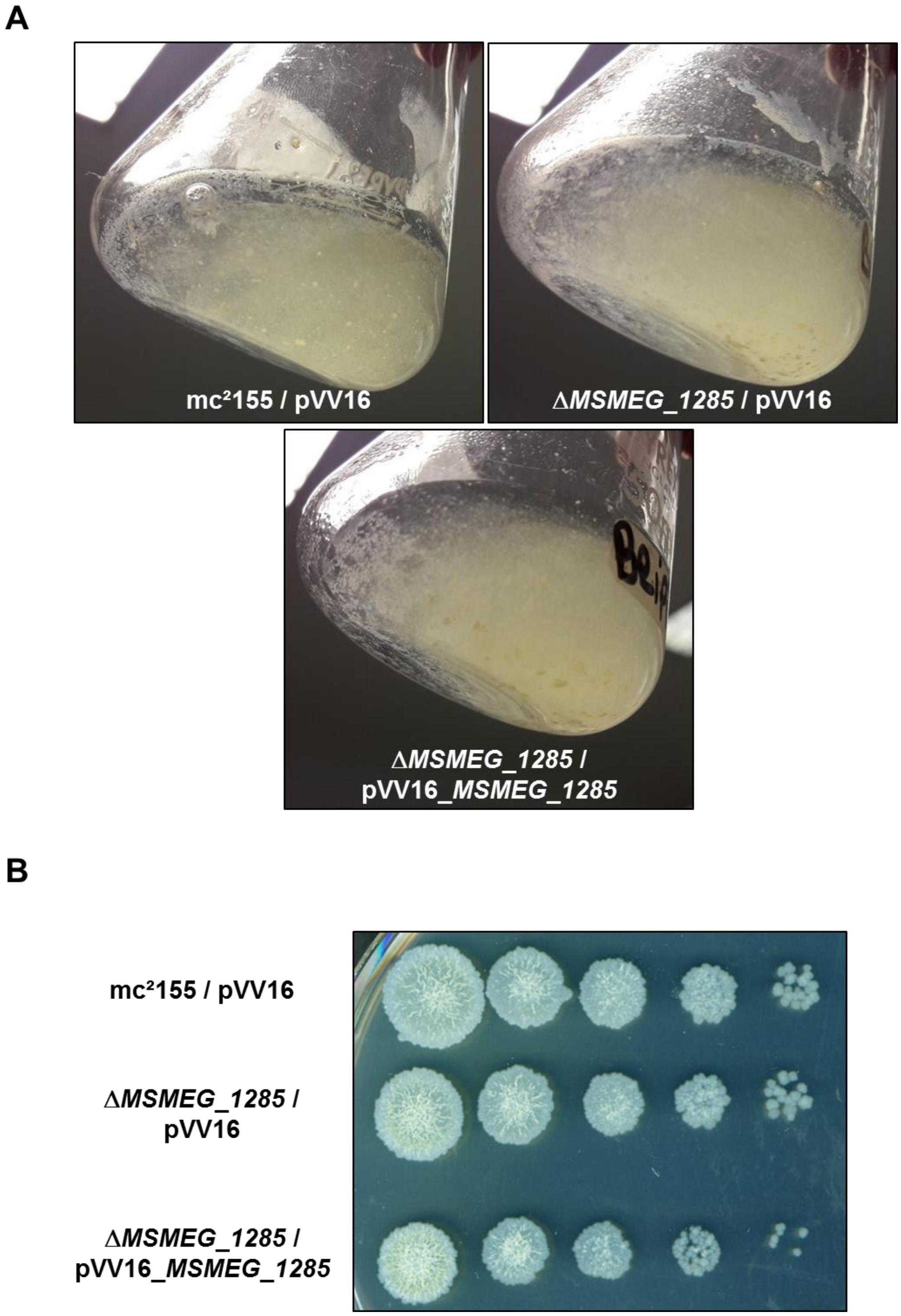

Supplementary Figure S4. Deletion of *MSMEG_1285* does not affect auto-aggregation and colony morphology of *Msmeg*. (**A**) Overnight cultures in 7H9 supplemented with OADC and without detergent of *Msmeg* mc²155, Δ*MSMEG_1285* mutant and the complemented strain; (**B**) Serial dilution of *Msmeg* mc²155 (upper series), the Δ*MSMEG_1285* mutant (middle series) and the complemented strain (lower series) on 7H11 agar plates.

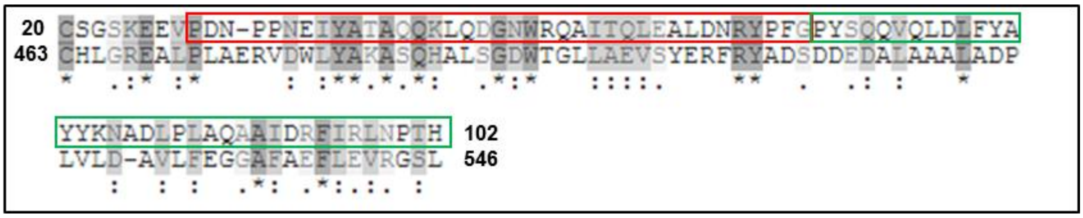

Supplementary Figure S5. Sequence alignment of the TPR1 and 2 from BamD and Rv0613c. Partial sequence alignment of BamD (first line) and Rv0613c (second line) using Clustal Omega. The red and green rectangles corresponds to the TPR1 and TPR2 domains of BamD, respectively. The numbers at the left and the right indicate the N-terminal and C-terminal amino acid positions, respectively, of the shown sequences. The grey boxes correspond to the level of similarity between the different amino acids.

Table S1. List of strains and plasmids used in this study.

Strains and plasmids	Genotype or description	Source or reference
E. coli TOP10	F^- *mcr*A Δ(*mrr-hsd*RMS-*mcr*BC) φ80*lac*Z ΔM15 Δ*lac*X74 *rec*A1 *ara*D139 Δ(*ara leu*)7697 *gal*U *gal*K *rps*L (StrR) *end*A1 *nup*G, used for cloning	Invitrogen
E. coli DHM1	F^- *gln*V44(AS) *rec*A1 *end*A *gyr*A96 *thi*-1 *hsd*R17 *spo*T1 *rfb*D1 *cya*-854	[1]
Msmeg mc^{2}155		ATCC 700084
Msmeg mc^{2}155 / pVV16	*Msmeg* mc^{2}155 containing empty vector pVV16	This work
Msmeg mc^{2}155 / pVV16_*MSMEG_1285*	*Msmeg* mc^{2}155 overproducing MSMEG_1285	This work
Msmeg mc^{2}155 / pMV361	*Msmeg* mc^{2}155 containing empty vector pMV361	This work
Msmeg mc^{2}155 / pMV361_*hbhA_EGFP*	*Msmeg* mc^{2}155 producing the fusion protein HBHA_EGFP	This work
Msmeg mc^{2}155 Δ*MSMEG_1285*	*Msmeg* mc^{2}155 with a deleted *MSMEG_1285* gene	This work
Msmeg mc^{2}155 Δ*MSMEG_1285* / pVV16	Deletion mutant Δ*MSMEG_1285* containing empty vector pVV16	This work
Msmeg mc^{2}155 Δ*MSMEG_1285* / pVV16_*MSMEG_1285*	Complemented strain of *Msmeg* mc^{2}155 Δ*MSMEG_1285*	This work
Msmeg mc^{2}155 Δ*MSMEG_1285* / pMV361	Deletion mutant Δ*MSMEG_1285* containing empty vector pMV361	This work
Msmeg mc^{2}155 Δ*MSMEG_1285* / pMV361_*hbhA_EGFP*	Deletion mutant Δ*MSMEG_1285* producing the fusion protein HBHA_EGFP	This work
pKT25	Multicopy *E. coli* vector encoding the T25 fragment (residues 1-224 of CyaA)	[2]
pKT25_*hbhA*	Multicopy *E. coli* vector encoding HBHA fused to the T25 fragment of CyaA	This work
pKT25_*hbhA*$_{1-109}$	Multicopy *E. coli* vector encoding truncated HBHA (amino acids 1 to 109) fused to the T25 fragment of CyaA	This work
pUT18C	Multicopy *E. coli* vector encoding the T18 fragment (residues 225-399 of CyaA)	[2]
pUT18C_*rv0613c*$_{314-550}$	Multicopy *E. coli* vector encoding truncated Rv0613c (amino acids 314 to 550) fused to the T18 fragment of CyaA	This work
pUT18C_*mmpL14*$_{519-749}$	Multicopy *E. coli* vector encoding truncated MmpL14 (amino acids 519 to 749) fused to the T18 fragment of CyaA	This work
pJSC347	Vector bearing hygromycin resistance cassette and used to generate allelic-exchange substrates	[3]

pJV53	Multicopy *E. coli* - mycobacteria shuttle vector allowing for the expression of phage recombinases	[4]
pVV16	Multicopy *E. coli* - mycobacteria shuttle vector, pMV261 derivative allowing for expression of C-terminal ^{6}His-tagged fusion proteins	[5]
pVV16_*MSMEG_1285*	pVV16 derivative used to produce His-tagged fusion of MSMEG_1285 in mycobacteria, used to complement *Msmeg* mc^{2}155 Δ*MSMEG_1285*	This work
pMV361	Monocopy *E. coli* - mycobacteria shuttle vector, *hsp60* promoter, integrative at *attB* site	[6]
pMV361_*hbhA_EGFP*	pMV361 derivative with *hbhA* and EGFP genes in translational fusion and under the control of the *hsp60* promoter	[7]

Table S2. List of primers used in this study.

Primers	5' to 3' sequence
pKT25_*hbhA*_dir	TATA**GGATCC**CGCTGAAAACTCGAACATTGATGAC (BamHI)
pKT25_*hbhA*_rev	TATA**GGTACC**TTCTGGGTGACCTTCTTGGC (KpnI)
pKT25_*hbhA*$_{1-109}$_rev	TATA**GGTACC**CGCAGCCGCTCTAGAGCGG (KpnI)
pVV16_*MSMEG1285*_dir	TATA**CATATG**GCCACCGTGACCGACGC (NdeI)
pVV16_*MSMEG1285*_rev	TATA**AAGCTT**GTCCAACCCCAACGCCGCG (HindIII)
Seq_pUT18C_dir	GAGCGGACGTTCGAAGTTCTC
Up_*MSMEG1285*_dir	TATATA**ACTAGT**GGCCGTATGCGCGGCGCG (SpeI)
Up_*MSMEG1285*_rev	TATA**AAGCTT**CCAAATCGCCCTCGGCCGC (HindIII)
Down_*MSMEG1285*_dir	TATATA**TCTAGA**CTACGTGCGCGCCGACGAC (XbaI)
Down_*MSMEG1285*_rev	TATA**AGGCCT**GGCTTTTCGGCTCACCGACG (StuI)
Seq_Δ*MSMEG1285*_1	GAGGTGCGCCAATATCTGCTG
Seq_Δ*MSMEG1285*_2	GCGCCATCGTGCAGGCCTG
Seq_Δ*MSMEG1285*_3	CAGGACCTGCAGGCATGCAAGC

References

1. Karimova, G.; Dautin, N.; Ladant, D., Interaction network among Escherichia coli membrane proteins involved in cell division as revealed by bacterial two-hybrid analysis. *J Bacteriol* **2005,** 187, (7), 2233-43.
2. Karimova, G.; Ullmann, A.; Ladant, D., Protein-protein interaction between Bacillus stearothermophilus tyrosyl-tRNA synthetase subdomains revealed by a bacterial two-hybrid system. *J Mol Microbiol Biotechnol* **2001,** 3, (1), 73-82.
3. Sambandamurthy, V. K.; Wang, X.; Chen, B.; Russell, R. G.; Derrick, S.; Collins, F. M.; Morris, S. L.; Jacobs, W. R., Jr., A pantothenate auxotroph of Mycobacterium tuberculosis is highly attenuated and protects mice against tuberculosis. *Nat Med* **2002,** 8, (10), 1171-4.
4. van Kessel, J. C.; Marinelli, L. J.; Hatfull, G. F., Recombineering mycobacteria and their phages. *Nat Rev Microbiol* **2008,** 6, (11), 851-7.
5. Jackson, M.; Crick, D. C.; Brennan, P. J., Phosphatidylinositol is an essential phospholipid of mycobacteria. *J Biol Chem* **2000,** 275, (39), 30092-9.
6. Stover, C. K.; de la Cruz, V. F.; Fuerst, T. R.; Burlein, J. E.; Benson, L. A.; Bennett, L. T.; Bansal, G. P.; Young, J. F.; Lee, M. H.; Hatfull, G. F.; et al., New use of BCG for recombinant vaccines. *Nature* **1991,** 351, (6326), 456-60.
7. Raze, D.; Verwaerde, C.; Deloison, G.; Werkmeister, E.; Coupin, B.; Loyens, M.; Brodin, P.; Rouanet, C.; Locht, C., Heparin-binding Hemagglutinin Adhesin (HBHA) is involved in intracytosolic lipid inclusions (ILI) formation in mycobacteria. Manuscript in preparation.
